# Computational Burden Resulting from Image Recognition of High Resolution Radar Sensors

**DOI:** 10.3390/s130x0000x

**Published:** 2013-04-22

**Authors:** Patricia López-Rodríguez, Raúl Fernández-Recio, Ignacio Bravo, Alfredo Gardel, José L. Lázaro, Elena Rufo

**Affiliations:** 1 National Institute for Aerospace Technology (INTA), Torrejón de Ardoz, 28850 Madrid, Spain; E-Mail: fernandezrr@inta.es; 2 Department of Electronics, University of Alcalá, Alcalá de Henares, 28871 Madrid, Spain; E-Mails: ibravo@depeca.uah.es (I.B.); alfredo@depeca.uah.es (A.G.); lazaro@depeca.uah.es (J.L.L.); elena.rufo@depeca.uah.es (E.R.)

**Keywords:** high resolution radar, radar imagery, ISAR, target recognition, computational burden, NCC

## Abstract

This paper presents a methodology for high resolution radar image generation and automatic target recognition emphasizing the computational cost involved in the process. In order to obtain focused inverse synthetic aperture radar (ISAR) images certain signal processing algorithms must be applied to the information sensed by the radar. From actual data collected by radar the stages and algorithms needed to obtain ISAR images are revised, including high resolution range profile generation, motion compensation and ISAR formation. Target recognition is achieved by comparing the generated set of actual ISAR images with a database of ISAR images generated by electromagnetic software. High resolution radar image generation and target recognition processes are burdensome and time consuming, so to determine the most suitable implementation platform the analysis of the computational complexity is of great interest. To this end and since target identification must be completed in real time, computational burden of both processes the generation and comparison with a database is explained separately. Conclusions are drawn about implementation platforms and calculation efficiency in order to reduce time consumption in a possible future implementation.

## 1. Introduction

Radar systems are key components in military and civilian schemes. Different applications have emerged since World War II related to this kind of sensor. A **R**adio **A**id to **D**etection **A**nd Ranging (radar [1]) is an electromagnetic sensor used for the detection and location of energy scattering objects. These systems not only have the ability to detect targets and show their position, but also to generate images and carry out certain electronic attack tasks, among many other applications. The basic principle of radar sensors is based on the time needed by the emitted electromagnetic wave to reach a target and back.

**Figure 1.**
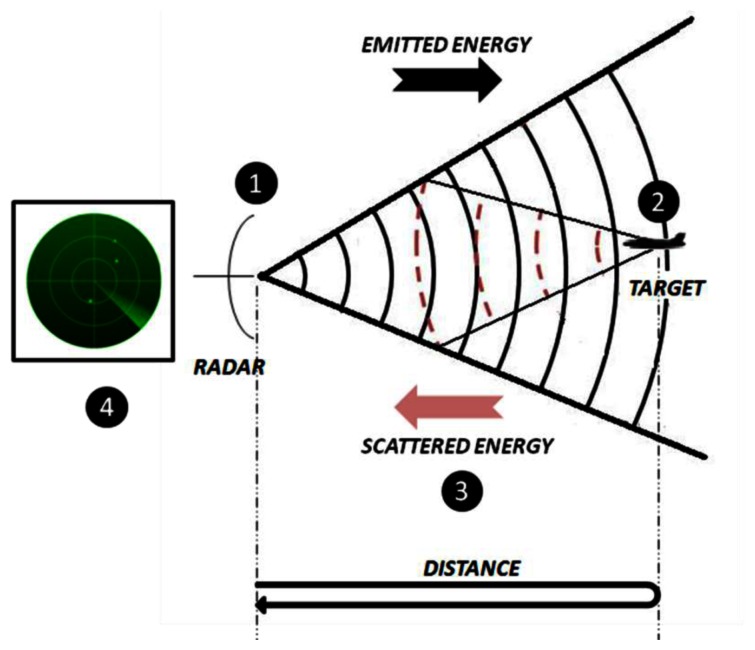
Basic principle of a radar system.

This principle is depicted in Figure 1, and can be divided into the following phases [1,2]:

1. The radar emits an electromagnetic energy which travels through space.
2. If the transmitted energy hits a target, it will be scattered in all directions.
3. Part of the scattered energy travels back to the radar and it will be sensed by the receiving antenna.
4. In the receiver, energy is amplified and with the aid of signal processing techniques the presence of a target may be determined. Not only the existence of targets can a radar detect but also other parameters such as its range, its radial velocity, or even the shape and size of the target if the radar has enough resolution to resolve closely spaced points within a target.

In the last decades, radar technology has experienced a change in its focus. Whereas in the beginning only the detection and tracking of targets was necessary, with the advance of technology the need to obtain higher spatial resolution has emerged. Consequently, radars have evolved into more flexible devices with the ability to generate high resolution imagery for mapping purposes or target identification [3]. Radars are the most suitable sensors for a rapid and reliable recognition of targets as they can operate in scenarios where visibility is very poor, such as bad weather conditions, smoky and dusty environments, *etc.* Their ability to resolve targets at a long range as well as their operation under any weather conditions makes them differ from other sensors like thermal or optical ones [2].

Target recognition using radar sensors can be divided into two techniques: cooperative and non-cooperative [1]. Cooperative techniques, known as identification friend or foe (IFF), require the communication between target and radar, while non-cooperative techniques, so-called non-cooperative target identification (NCTI), do not establish any communication with them but rely on the comparison of the measured targets with a reference database. This database is usually populated with actual target measurements obtained in scheduled measurement campaigns [4]; however, it implies the collection of information from a great number of flying targets in different aspect angles and configurations and even so, the main problem lies in the fact that not all existing aircrafts may be measured. For this reason, other methods have been deployed to populate the database. These methods include measurements in anechoic chamber and electromagnetic simulations [5]. The latter is of great interest due to its low cost and the simplicity of obtaining a vast number of CAD aircraft models for electromagnetic simulations.

In this paper a target recognition methodology based on high resolution radar imagery is presented. Algorithms related to high resolution radar image creation and the problems found are introduced, as well as a target recognition methodology based on image cross correlation. High resolution radar image generation and target recognition processes are complex and time consuming. The goal of a NCTI system is the reliable recognition of targets in real time; therefore, studies on the computational burden of the whole process are of great interest. These studies will make it easy to identify the computationally critical points of the system in order to previously choose an implementation platform that could perform these operations efficiently. Accordingly, the computational burden of the proposed system is revised distinguishing the complexity of image generation from the complexity of target recognition. With these results conclusions about implementation platforms and calculation efficiency are drawn in order to reduce time consumption in a possible future implementation.

The article is organized as follows: Section 2 introduces high resolution radars as image sensors bringing into focus inverse synthetic aperture radars (ISAR). Section 3 presents the methodology used in this study for ISAR image generation from actual flying aircrafts data and its recognition, based on the previous work by [6]. The methodology presented requires complex computations implying a high computational burden as it is explained in Section 4. Finally, Section 5 discusses the results and conclusions, calling for further work and research in the area.

## 2. High Resolution Radars

**Figure 2.**
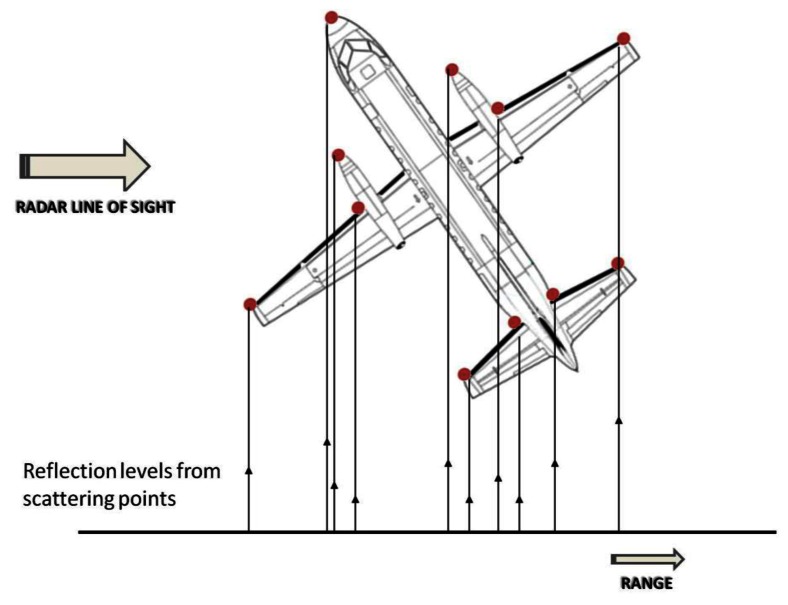
Example of scattering centers in a target.

To high resolution radars (HRRs) targets appear as comprised of individual scattering points, also called *scattering centers*, *backscatter sources* or *scatterers* [7]. Figure 2 shows an example of these scattering centers projected on the radar line of sight direction. At a given viewing angle (*target aspect angle*), each *scatterer* reflects energy at a certain amplitude and phase. High resolution radars have the ability to discern the different scattering centers of a target in both the propagation and the transversal direction of the transmitted energy; being able, therefore, to identify the geometry of a target. Thus, resolution of these radars is defined in two dimensions, on the one hand there is the *slant-range* resolution which depends on the radar bandwidth and is defined as the ability to resolve *scatterers* in the direction of the radar line of sight; on the other hand, there is the *cross-range* resolution which depends on the wavelength of the emitted signal and the angular sweep made during the illumination time. *Cross-range* resolution is defined as the ability to resolve *scatterers* in the normal direction to the plane containing the radar line of sight and the target rotation angle.

There exist mainly two different types of HRR: *synthetic aperture radars* (SAR) and *inverse synthetic aperture radars* (ISAR). Both make use of the relative motion of target and radar to achieve high resolution in the cross-range direction.

SAR radars achieve high resolution in the cross-range dimension by taking advantage of the motion of the vehicle carrying the radar to synthesize the effect of a large antenna aperture [2,7,8]. These sensors are usually used for imaging the Earth's surface to provide maps for military or civilian reconnaissance, measurements of sea state, geological and mineral explorations and other sensing applications. SAR requires coherence between signals and the means necessary for the storing and subsequent processing of the received echoes. ISAR imagery is based on the same principle as SAR imagery, but in contrast it is the target rotational motion which will generate the necessary information for obtaining the image while the radar remains stable [8,9].

### 2.1. Inverse Synthetic Aperture Radar

High resolution radar imagery obtained by ISAR radars can be 1-dimensional or 2-dimensional. On the one hand, 1D images present the *scatterers* of a target projected on the dimension of the radar line of sight (LOS) that is in *slant-range*, or the *scatterers* of a target projected on the *cross-range* dimension. 1D images projected on *slant-range* are called *high resolution range profiles (HRRP)* while those projected on the *cross-range* dimension are called cross-range profiles [7,9].

Usually the stop & go assumption is held, which means that the target is assumed stationary during the transmission and the reception of a pulse. Sometimes however, this statement cannot be assumed valid because the pulse repletion time is too long or because the target moves very fast. In such cases an autofocusing technique is also needed to form HRRP [10,11]. The cross-range profiles are obtained by exploiting the target motion with respect to the radar and by using the aspect angle changes to synthesize the aperture. Obviously an auto-focusing step is needed first. This paper works with range profiles (HRRP) instead of cross-range profiles and the stop & go approximation is assumed to be valid so no autofocusing technique is needed to obtain HRRP.

**Figure 3.**
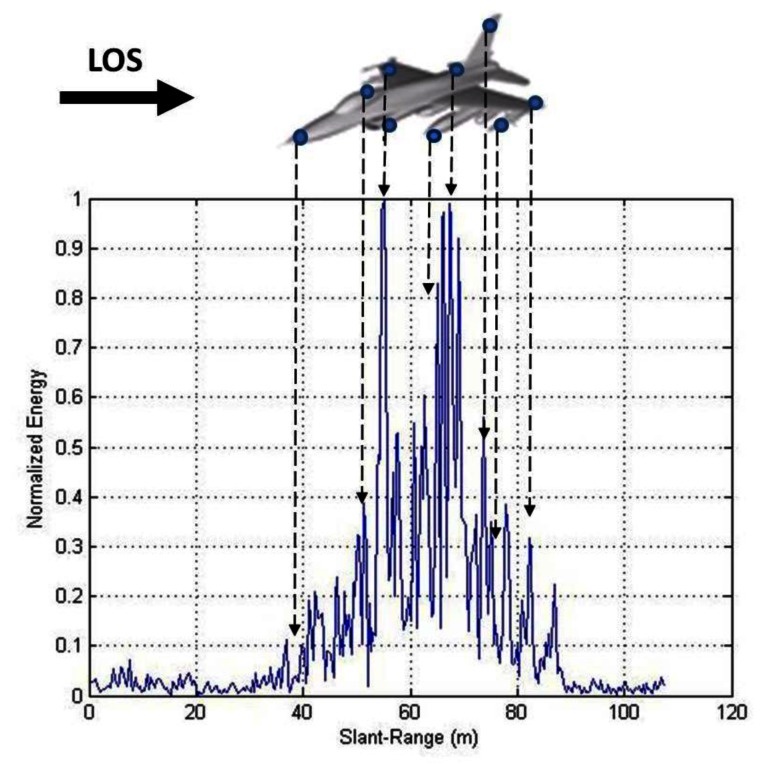
High resolution range profile.

HRRP represent the energy reflected by every *scatterer* in a moving target as a function of distance. Each profile is comprised of *range bins* that can contain energy from different *scattering centers*. Figure 3 depicts how high resolution range profiles present the energy reflected by the *scatterers* of a target in the dimension of the radar line of sight. Signal processing needed to obtain the HRRPs of a target is not very complex; however, they are very sensitive to the target viewing angle (aspect angle) due to occlusion of *scatterers* or other unwanted effects such as speckle or rotational range migration (RRM) [4].

The resolution of a range profile is dependent on the bandwidth of the emitted signal; the shorter the emitted pulse, the wider the bandwidth and the finer the resolution [2,7]. Unfortunately, there are limitations in the reduction of the width of the emitted pulse since it is limited by the energy the radar is capable of transmitting. Most radars are not able to transmit the power needed to achieve high resolution with a pulse waveform. Nevertheless, the *pulse compression* [8] technique allows radars to obtain high resolution using long pulse widths. This technique consists of modulating the frequency of the emitted waveform along the total pulse width. The receiver is in charge of the quadrature demodulation of the received signal using a matched filter to maximize the Signal-to-Noise Ratio (SNR). Typical waveforms used in pulse compression techniques are *chirp* [12] and *stepped-frequency* [13]. Radars using pulse compression technique sense the total radar returns in the frequency domain; hence, HRRPs are obtained by applying an inverse Fourier transform to the radar complex returns [9].

**Figure 4.**
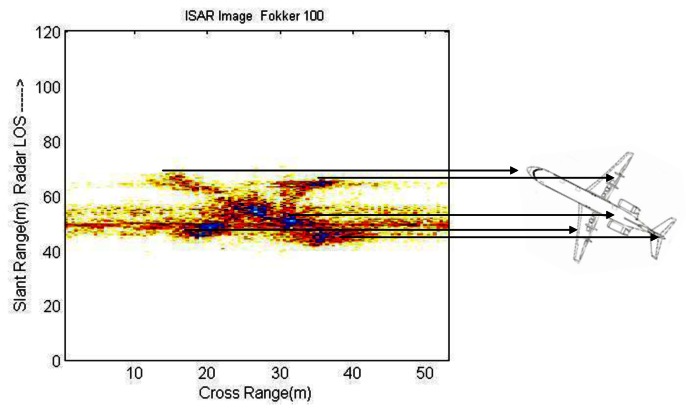
Scattering centers in an ISAR image.

On the other hand, 2D images, named ISAR images, represent the geometry of a target in both *slant*- and *cross-range*. ISAR images contain information of consecutive HRRPs with small angular variation; these images display the distribution of scattering centers within a target in the perpendicular direction of the target's rotation plane [14]. Figure 4 depicts the fact that ISAR images present the scattering centers of a target in two dimensions. The aircraft displayed in this figure correspond to a Fokker-100.

**Figure 5.**
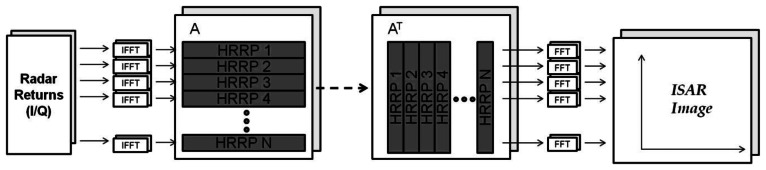
RDA Algorithm.

Signal processing needed to achieve ISAR images is complex and implies higher computational burden than that needed for the generation of HRRPs. There are several methods used in literature to form ISAR images including back-projection methods [15] or range-instantaneous Doppler algorithms (RID), such as Radon-Wigner transform (RWT) method [16], joint time-frequency analysis method [17], reassigned smoothed pseudo Wigner-Ville distribution [18], fractional Fourier Transform [19], *etc.* The algorithm used in this paper for the creation of ISAR images is called range-Doppler algorithm (RDA) [7,9]. This technique is the most common since it is the simplest one. It mainly consists in the application of a double Fourier transformation; first, an inverse Fourier transform is applied to the quadrature demodulated data (I/Q samples) in order to obtain a matrix filled with high resolution range profiles and second, a Fourier transform is applied to every range bin of these profiles in order to acquire information of the *scatterers* in the *cross-range* dimension. The basic approach of this algorithm is depicted in Figure 5 where A denotes the profiles matrix and A^T^ is its transpose.

Target motion with respect to the radar makes it possible to achieve ISAR images; nevertheless, not every movement is desired and this may cause blurring in the obtained images. In order to avoid this defocusing, motion compensation techniques must be applied [9,20].

Generally speaking, target motion can be decomposed into translational and rotational [14,21,22]. In order to get focused images both the translational and rotational motion must be compensated. Translational motion causes consecutive HRRPs to be misaligned, so in order to compensate it an alignment of profiles must be completed, this procedure is also called *range bin alignment*. In addition to profile alignment, a *phase adjustment* procedure must be applied in order to refer every measurement to the same origin [9]. In the past decades, translational motion compensation has been of great interest and now it has become a well-established technology. Range bin alignment methods are rather standard, including centroid tracking [23,24], envelope correlation [14], contrast/entropy based methods [25], prominent point processing or dominant scatterer algorithm [26], *etc.*

On the other hand, rotational motion causes *motion through resolution cells* (MTRC) [27] which produces the *scatterers* to move from bin to bin in *slant*- or *cross-range*. However, it can be ignored provided that the target is small or the required resolution is coarse [22].

Many algorithms have been proposed in the literature for motion compensation in ISAR imaging. What is presented here is the computational complexity analysis of a combination of translational motion compensation methods (envelope correlation and dominant scatterer algorithm) in order to get a focused ISAR image. The driving idea is to achieve an affordable processing chain, in terms of computational burden which is the mandatory requirement for a future possible implementation in real time.

## 3. ISAR Generation and Target Recognition System

**Figure 6.**
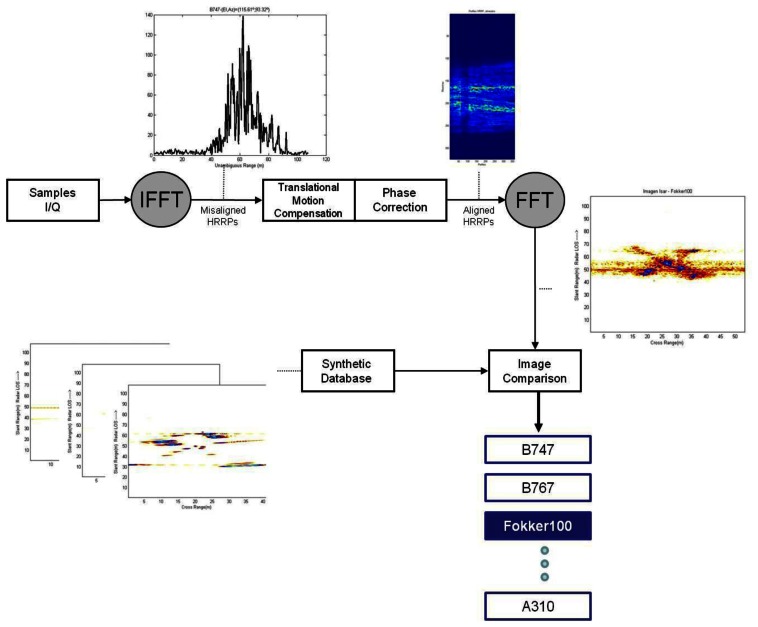
System identification flowchart.

The complete system under study is implemented in Matlab® (R2008a) and consists, firstly, of the generation of an ISAR image from a dataset of flying aircrafts. To that end, motion compensation of high resolution range profiles must be implemented. Secondly, after an ISAR image is obtained, the comparison with a database of ISAR images is carried out with the final purpose of aircraft recognition. This database is populated with ISAR images generated synthetically with electromagnetic software. Figure 6 depicts the flowchart of this procedure.

### 3.1. Data Set

The use of actual data in the generation of ISAR images is of great importance since it is possible to obtain realistic images that could not be obtained by any other means. However, it must be noted that actual data is not usually accessible and not easy to measure since the use of high level technology resources is required.

The North Atlantic Treaty Organization (NATO) performs different activities under its Research and Technology Organization (RTO). Data used in this work comes from the ORFEO civilian airliner measurement campaign, held in 1995 and obtained with the FELSTAR radar. FELSTAR is a stepped-frequency S-band radar owned by TNO-FEL and located in The Hague, The Netherlands [28]. This measurement campaign was carried out as part of the RTO-SET-040 Task Group activity and up to 17 different civilian aircrafts were measured as targets of opportunity. By using actual data from the ORFEO campaign and applying the RDA algorithm explained in previous sections, ISAR images of different civilian aircrafts are obtained.

### 3.2. ISAR Image Formation

**Figure 7.**
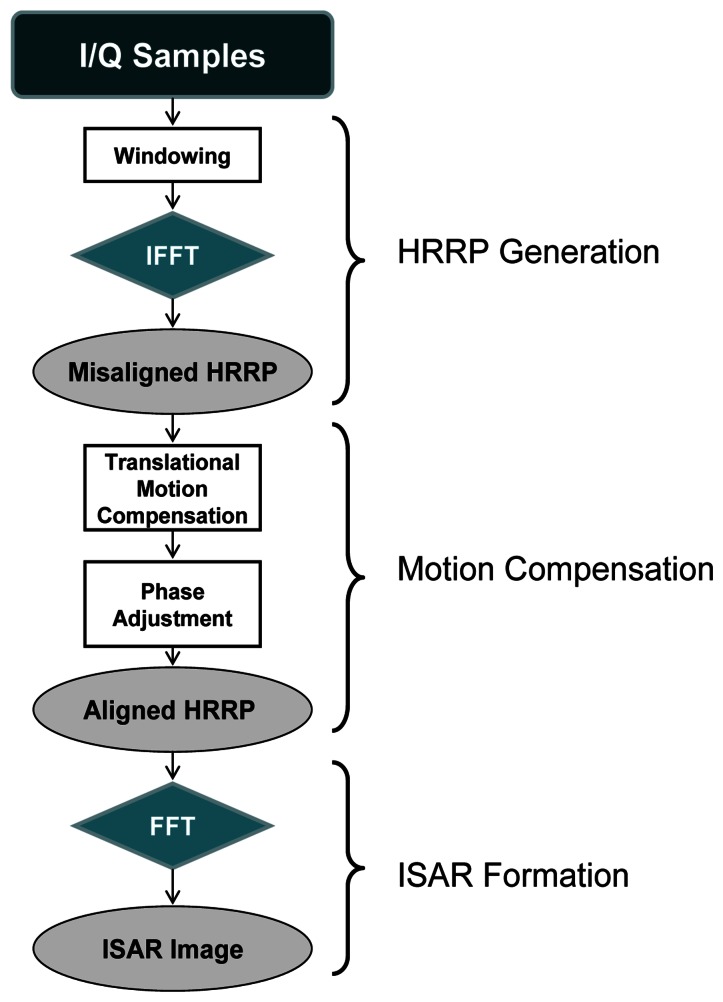
ISAR image formation flowchart.

This section describes the algorithm used for the generation of an ISAR image from actual data using Matlab®; this procedure is based on the flowchart in Figure 7. As mentioned above, for the generation of a focused ISAR image, in addition to the implementation of RDA the implementation of a motion compensation method is also necessary.

In order to acquire a more focused image a Hamming window [4,29] is first applied to the samples since it reduces sidelobes in 43 dB. In case a Hamming window was not employed, with the inverse Fourier transform needed to obtain the profiles, a rectangular window would be automatically applied which has high sidelobes that can produce the occlusion of scattering centers [30].

**Figure 8.**
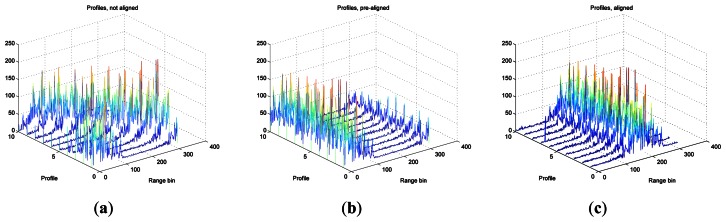
Profile alignment process; (**a**) initially misaligned HRRP; (**b**) pre-aligned HRRP; (**c**) aligned HRRP after translational motion compensation.

The next step after windowing and application of inverse Fourier transformations (using the IFFT algorithm) is the translational motion compensation of the obtained HRRPs. To align the profiles an algorithm based on the envelope correlation method [14,31] is applied first. In the case covered in this article, a reference profile is first established defined as a sum of six aligned profiles after applying correlation between them. The remaining profiles will then be aligned by correlating them to the reference one. Note that the reference profile must be updated after a new profile is aligned by including the new one to the reference profile and discarding the oldest one. After pre-alignment using envelope correlation (coarse alignment), a fine alignment is applied. This fine-alignment comprises three steps: first, a prominent *scatterer* must be selected; second, profiles are re-aligned by tracking the prominent *scatterer* along the profiles matrix; to do so, profiles maxima are found within a small band from the prominent *scatterer* and, when necessary, profiles are realigned. Finally, phase adjustment is carried out by using the dominant scatterer algorithm (DSA) [26,32] where the phase of the dominant *scatterer* previously selected is subtracted from the phase of the rest of the profiles already aligned. Figure 8 shows the whole translational motion compensation process; Figure 8(a) presents the initial 10 HRRPs of a measured Boeing-767, and as can be seen, these profiles are completely misaligned. By employing envelope correlation profiles are pre-aligned as shown in Figure 8(b). Lastly, after phase adjustment profiles are finally aligned as in Figure 8(c).

**Figure 9.**
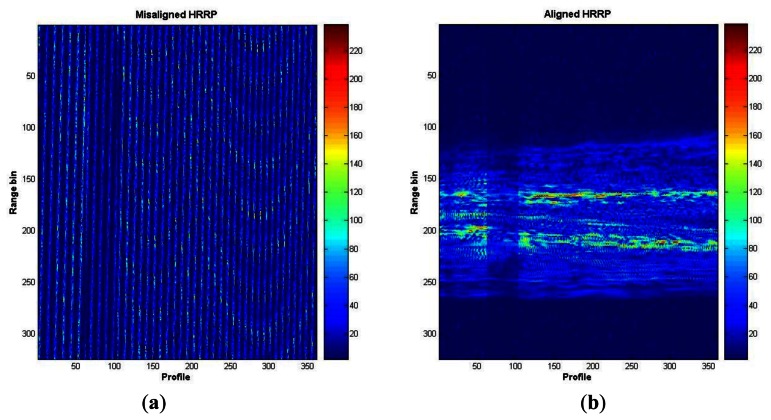
Profile alignment processm—2D; (**a**) initially misaligned HRRP; (**b**) aligned HRRP after translational motion compensation.

Figure 9(a) shows the initially misaligned profiles as in Figure 8(a), but in a 2D plot. This figure presents the whole set of profiles of a measured Boeing-767 in the ORFEO campaign. Figure 9(b) shows the resulting aligned profiles as in Figure 8(c) in 2D. With this last figure it is easy to observe the evolution from a misaligned set of HRRPs to an aligned set.

Regarding rotational motion compensation [9,33], it has already been noted that it would only be necessary when the resolution needed is very fine or target rotation is very high. Neither case is present in this study; hence, this step is omitted.

**Figure 10.**
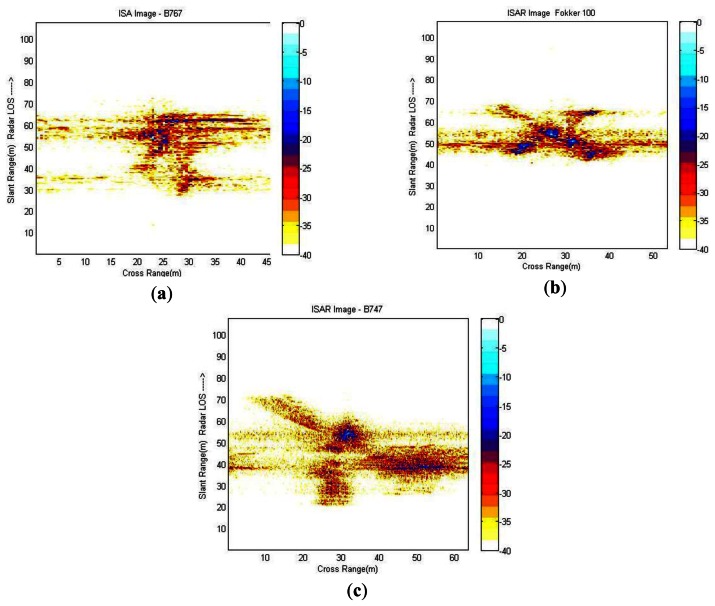
ISAR images obtained from the ORFEO measurement campaign; (**a**) ISAR image of a Boeing 767; (**b**) ISAR image of a Fokker 100; (**c**) ISAR image of a Boeing 747.

Finally, application of Fourier Transformation (using the FFT algorithm) to the range bins of the aligned profiles is applied and an ISAR image is obtained. Examples of different ISAR images obtained by means of the procedure described in this section are displayed in Figure 10. As expected, ISARs obtained are not of great quality if compared to a video or an IR sensor image although they have enough quality to discern the existence of an aircraft with certain geometry and dimensions.

As observed in Figure 10, a blurred band exists approximately in the middle of every image. This blurring is due to the fact that these images were produced using actual data and some noise and clutter could not have been completely removed. This will probably affect in the identification stage, resulting in a degradation of the final result.

### 3.3. ISAR Image Comparison (Target Recognition)

**Figure 11.**
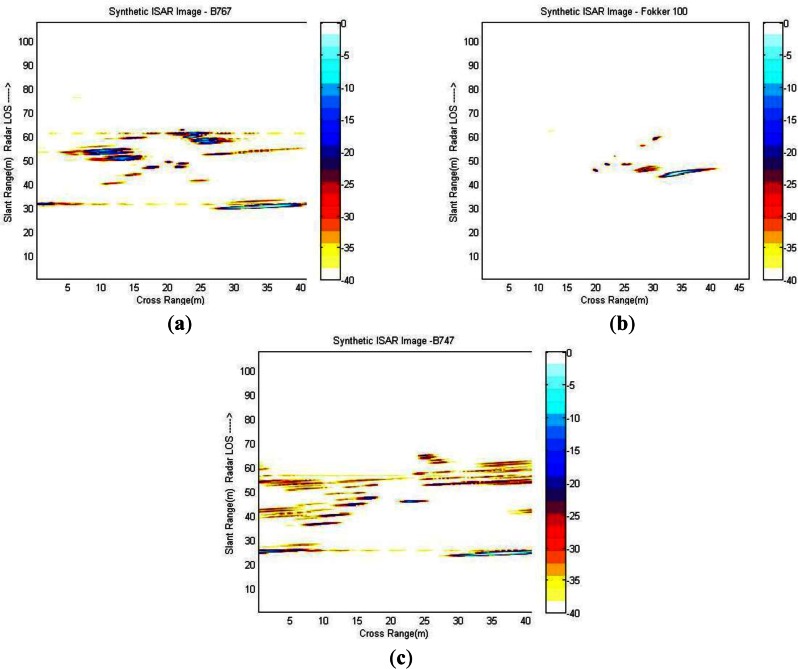
Examples of ISAR images populating the synthetic database; (**a**) Synthetic ISAR image of a Boeing 767; (**b**) Synthetic ISAR image of a Fokker 100; (**c**) Synthetic ISAR image of a Boeing 747.

Target recognition is accomplished by applying a template-matching technique where targets are recognized based on the template that best matches the reconstructed ISAR images. The recognition is carried out by comparing ISAR images obtained from actual data to a database populated with synthetic ISAR images, that is to say images obtained with electromagnetic software. FASCRO is the tool employed in this paper in order to generate the synthetic images that will populate the database. The software is based on the work by [34,35]. Its operation lies in a combination of two high frequency techniques, physical optics (PO) and physical theory of diffraction (PTD) applied to targets modelled as non-uniform rotational B-splines surfaces (NURBS) [36,37]. Figure 11 displays some of the synthetic images that populate the database.

As can be easily noticed, synthetic ISAR images are in many ways different from those obtained from actual data; the images obtained synthetically are much clearer; this is due to the fact that electromagnetic software runs an ideal scheme, without considering any noise or clutter. Synthetic ISAR images do not suffer from measurement noise, and also application of translational motion compensation to HRRPs is not needed for their generation. Moreover, all the aircrafts are considered PEC (*perfect electric conductors*) in the simulations and also CAD models are approximations of aircraft geometry. Thus, electromagnetic software cannot simulate all the effects present in a real environment. Additionally, for the construction of a good database of synthetic aircrafts the image projection plane (IPP) must be taken into account since the target reflectivity is strongly dependent on the aircraft aspect angle and can affect the recognition process. Moreover, the estimation of the angular velocity of target rotation should be carried out since cross-range scaling of the ISAR image depends on it. In [38,39] an attempt to solve the question of building a robust database can be found taking into account the image projection plane. In [40] an iterative method to estimate the angular parameters of non-cooperative targets using the estimates of the range and radial velocity of two prominent *scatterers* as inputs is addressed.

In the study presented here the flight plans of the different aircrafts are known and the database has been built according to them. This implies that the image projection plane and the angular velocity of the targets in the database used in this paper for recognition are the same as the ISAR images generated from actual data; this means that their estimation is not necessary. However, in a real application of non-cooperative target recognition flight plans are unknown and the aspect angle of the aircrafts as well as their angular velocity should be estimated. Consequently, the database should be populated with ISAR images of aircrafts in different aspect angles and trajectories and the ISAR images of the targets should only be compared to those with the same resolution and image projection plane in order to reduce computational burden. The proposed template-matching technique to compare ISAR images is the normalized cross-correlation between them [41–43] although there is no generally accepted way of performing this task. Normalized cross correlation (NCC) is one of the most robust measures for determining similarity between points in two or more images providing an accurate result. However, this method can be computationally intense, especially for large images [44]. Equation (1) presents the formula of the NCC:

(1) $$r=\frac{\overset{M-1}{\underset{m=0}{\text{∑}}}\overset{N-1}{\underset{n=0}{\text{∑}}}\left(A_{mn}-\bar{A})\;(B_{mn}-\bar{B}\right)}{\sqrt{\;[\overset{M-1}{\underset{m=0}{\text{∑}}}\overset{N-1}{\underset{n=0}{\text{∑}}}{\left(A_{mn}-\bar{A}\right)}^{2}]\;\left[\overset{M-1}{\underset{m=0}{\text{∑}}}\overset{N-1}{\underset{n=0}{\text{∑}}}{\left(B_{mn}-\bar{B}\right)}^{2}\right]}}$$

where *A* and *B* are images of size *N* × *N* and *M* × *M* respectively, and *A̅*) and B̅ denote their mean value. In the present case, both images have the same size. Table 1 summarizes some of the results obtained using normalized cross-correlation for target identification.

Results of the identification method show low correlation between images, even though the highest value is obtained for the aircraft to be recognized. The reason why these results are obtained lies in the fact that synthetic images are much clearer than those obtained from actual data. To improve these correlation results further image processing should be applied to either image set. Note that this additional image processing does not have to do with RDA or motion compensation but with noise/clutter rejection techniques; however, that is not the purpose of the present work but the study of the computational cost of the generation of ISAR images from actual data and the comparison with a database.

## 4. Computational Burden Results

One of the requirements in an automatic target recognition method is to obtain a result in real time. Real time can be considered as the time needed to process a result sufficiently rapid in order for the radar operator to be able to make decisions. For this purpose high performance devices are usually needed to achieve these time requirements.

Prior to the selection of a device to implement a system it is of high interest to study the computational burden by means of analyzing the order of magnitude of the calculations. Matlab^®^ Profiler [45] is an excellent tool for a preliminary study on computational cost, it was first developed to provide information for the debugging and optimization of code but it also provides information about execution time of functions, the number of times a function is called, computing time in CPU and even the memory consumed by each function. Consequently, a study of computational burden of both the generation and the comparison process is carried out using Matlab^®^ Profiler (R2008a) in order to identify critical computational points.

In the next subsection the computational complexity of ISAR image formation is studied, establishing for each stage in which the process can be decomposed into the number of operations needed. Finally, computational complexity of ISAR image comparison for target recognition is revised.

### 4.1. ISAR Generation Process Computational Cost

ISAR generation process, as noted in previous sections, is comprised of the subsequent stages:

● HRRP Generation
● Motion Compensation
● ISAR Formation

**Figure 12.**
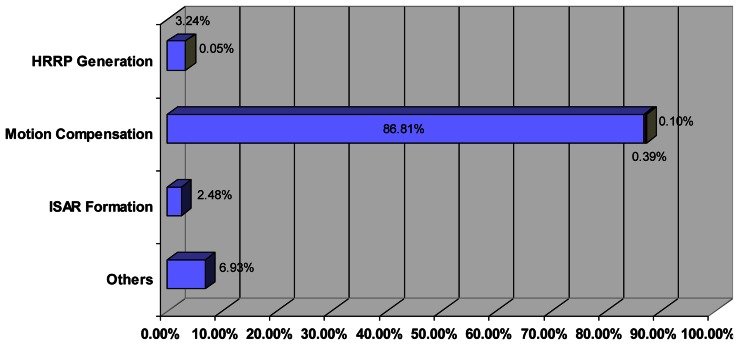
Time spent in the generation of an ISAR image.

Each stage comprises operations dependent on the number of high resolution range profiles (N) and the number of different frequencies in a burst of the transmitted stepped-frequency signal (M). According to the results given by Matlab® Profiler, Table 2 and Figure 12 show the operations needed and the percentage of time spent in each stage of the process. The operations grouped under “Others” in Figure 12 are those needed to plot images, load/save data or display Matlab® Profiler main window. The ISAR image generation process was run in an Intel Xeon @ 2.66GHz and 3.50 GB RAM PC and the average total time spent for the generation of an image of size 361 × 324 pixels (N = 361 profiles × M = 324 frequencies in a burst) was approximately 40 seconds.

According to Table 2 and Figure 12 the most expensive (computationally speaking) stage is the motion compensation, and more specifically the process of the range bin alignment which involves the realization of (N – 1) circular correlation of M real samples and needs an 86.81% of the total time.

Being *x* [*m*] and *y* [*m*] two profiles of M samples, circular cross correlation is accomplished by applying Equation (2), where the term *x* [*m*plus;*k*] *_M_* denotes *x* [*(m+k) mod M*], that is to say, the circular shift of *x* [*m*]:

(2) $$\phi_{xy}\left[k\right]=\frac{1}{M}\overset{M-1}{\underset{m=0}{\text{∑}}}x{\left[m+k\right]}_{M}\cdot y[m]$$

Analysis of Equation (2) reveals that for every circular correlation of M samples a total of M^2^ products, M·(M – 1) sums and M circular shifts are employed. These operations will be complex or real depending on the nature of data. In this particular case, operations needed for circular correlations in the range bin alignment stage are real. Moreover, the normalization of the correlation is not needed since all profiles will be scaled by the same factor and it will not have effect in the alignment. It should be noticed that another technique to perform correlation between signals is through the use of FFT, which converts correlations into a product of transforms.

The method revised here for range bin alignment, based on the envelope correlation, requires the creation of an initial reference profile made up of the first six aligned HRRPs. The alignment and creation of this reference profile implies the execution of five correlations out of the (N – 1) needed to accomplish the whole alignment process. Next step is the correlation with the following profile, the alignment with it and the update of the reference profile. This process will be repeated until all profiles are aligned, therefore, it will be repeated (N – 6) times. The update of the reference profile implies 5M sums. The total number of operations needed for range bin alignment of a total number of N profiles made up of M samples is summarized in Table 3.

From Table 3 it can be deduced that this operation has an order of magnitude of **O(N·M^2^)**. That means, for an image of size 361 × 324 pixels, 37,791,360 real products, 38,249,820 real sums and 117,360 circular shifts. With the implementation of the range bin alignment process in a parallel device, the computational burden could be reduced to a magnitude of order **O(N·M)**, or even further to a magnitude of **O(N)** in cases where the implementation device has enough resources.

This order of magnitude could not be further reduced, at least initially, since parallelization of more operations could not be applied due to the dependence of correlations. This means that only one correlation is done at a time because the reference profile must be computed before the next correlation can be executed.

Although FFTs/IFFTs have not been very time consuming operations compared to the range bin alignment stage, it should be noted that a high number of operations are also required. Matlab® FFT algorithm is based on a library called FFTW [46] which has a computational complexity of O(N·log_2_(N)), where N is the number of samples. Only if the magnitude of the range bin alignment was reduced to O(N), would FFT computational cost and alignment process cost be comparable.

### 4.2. ISAR Image Comparison (Target Recognition) Computational Cost

The method proposed to compare ISAR images is the normalized cross-correlation between them showed in Equation (1). It is worth noting that template-matching techniques like the one proposed here are computationally expensive since ISAR images are normally of large dimensions. To speed up the recognition process other approaches have been proposed in the literature based on the comparison between a set of features extracted from the ISAR image to be recognized and a database of features [38,39,47].

In the present case both images will be squared of the same size *N* × *N*, so by using normalized cross-correlation one can deduce that a computational complexity of order O(N^2^) is needed. Nevertheless, images are not centered at the same point so image matching should be additionally applied. This matching implies the increase of the computational complexity to an order of **O(N^4^)**, since additional N^2^ comparisons are executed, one per pixel image. This high burden leads to an average time of 10 minutes to complete one comparison of two images of size 256 × 256 in an Intel Xeon @ 2.66 GHz and 3.50 GB RAM PC; thus, recognition using normalized cross-correlation is clearly a bottleneck in the system and its computational complexity must be reduced. In order to do so, instead of a pixel by pixel matching, one of the images is shifted ′*g*′ discrete steps (pixels) in both the x and y dimensions while the other remains stable and the correlation is calculated for each position creating a correlation matrix. The following step is to find the highest correlation value in this matrix and to define a range around it in which normalized cross-correlation will be applied again by shifting images pixel by pixel.

**Figure 13.**
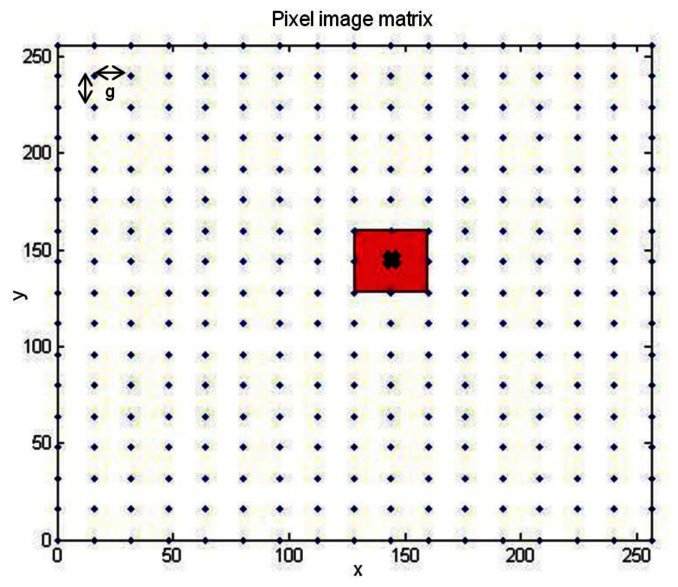
Shifts applied in ISAR image comparison (g = 16 pixels).

To clarify the process Figure 13 shows blue points as the initial shifts where the “X” presents the point of maximum correlation. In red is depicted the range in which the correlation will be applied shifting images pixel by pixel.

This image shifting based on the work in [48], implies the execution of *C* normalized cross-correlations instead of N^2^. In Equation (3)*N* denotes the image axis size and *g* is the shift applied (16 or 32 pixels depending on the image size):

(3) $$C={\left(\frac{N}{g}+1\right)}^{2}+{\left(2g+1\right)}^{2}$$

**Figure 14.**
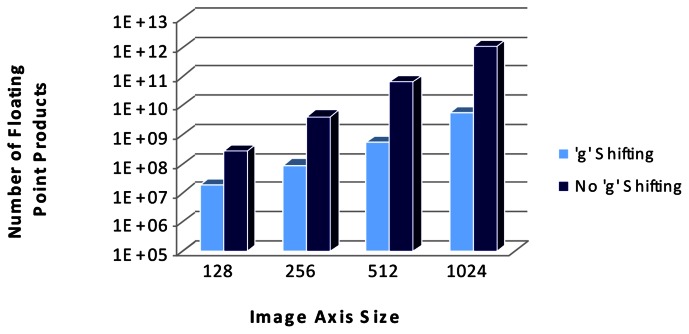
Computational complexity of normalized cross correlation.

Hence, by using this shifting scheme to carry out image comparisons the computational burden is reduced from an order of magnitude of **O(N^4^)** to an order of **O(C·N^2^)**. As an example, for the comparison of two 256 × 256 images with a shift of g = 16 pixels, from Equation (3) the number of correlations needed to obtain a result is C = 1,378 whereas if shifting was not applied a total number of N^2^ = 65,536 correlations would be executed. Figure 14 compares the order of magnitude in a logarithmic scale of normalized cross-correlation when image shifting method is applied or not, with “*g*” being 16 pixels.

Another relatively efficient way of calculating the NCC is by using the FFT to compute the numerator of Equation (1), however, the denominator of the NCC in Equation (1) does not have a correspondingly efficient frequency domain expression [44,41]. In order to simplify computation of the denominator a sum-table of precomputed data acting as a lookup table should be used, resulting in a reduction of the computational complexity. Other methods to compute the NCC include the utilization of basis functions to approximate the image template [41].

In the method studied in this paper, as Figure 14 shows, a great reduction in the number of operations executed is achieved with this approach and hence, running time has been lowered from an average of 10 minutes to an average of 25 seconds. However, by selecting a more suitable implementation platform operations could be parallelized and execution time could be further reduced.

As already stated, in the study presented in this paper the estimation of the angular velocity of the targets is not done since the simulated aircrafts have the same trajectories than the actual measurements. However, in a real application of non cooperative target recognition angular velocity of targets should be estimated resulting in an increase of the computational complexity. As an example of how much the computational burden could be increased can be found in [40] where an iterative process to estimate the angular parameters of an aircraft is presented. That iterative method is of order O(L^3^) per iteration, with L belonging to the interval (50,150) and the iterations being a maximum of 15. Hence, considering the estimation of the angular parameters is needed, an additional computational burden of O(L^3^) should be added to the order of O(C·N^2^) found in the realization of the image comparison. However, the order of magnitude in the estimation of the angular parameters is lower than the computational burden of the ISAR image comparison based on the NCC studied in this paper.

## 5. Conclusions

The use of radar sensors for non-cooperative target identification purposes is of great interest in civilian and military schemes. To this end a methodology for target recognition from ISAR images and the signal processing needed has been presented. It is always interesting the study of the computational complexity of any system with the aim of selecting the best implementation platform to achieve system requirements. In order to find the critical points in the target recognition system proposed in this paper, computational burden of ISAR image generation and comparison with a synthetic ISAR image have been analyzed using Matlab^®^ Profiler (R2008a). Results have revealed two critical points in the system presented here. On the one hand, in the ISAR generation process the bottleneck has been found in the profiles alignment. This alignment was based on the envelope correlation algorithm and had a computational complexity of O(N·M^2^), with N and M being the number of profiles and the number of frequencies in a stepped-frequency waveform respectively. On the other hand, in the target recognition process the bottleneck is found in images cross-correlation; it has been proved that by applying a certain shifting grid the computational complexity of the normalized cross-correlation could be reduced from O(N^4^) to O(C·N^2^) with the corresponding decrease in execution time.

According to the results obtained the main bottlenecks of the whole system lie in the high amount of correlations needed in both the alignment and recognition procedures. These operations can be decomposed into sums and products that can be efficiently executed in high performance parallel devices due to their high speed, internal resources and parallel execution. In conclusion, tools like programmable logic devices (FPGAs) or GPUs could be good candidates to implement and perform the system presented in this paper in real time with the additional advantage of fast reconfiguration and low cost.

Nonetheless, further investigation is being considered in the identification process where the resemblance between generated ISAR images and database images is very low resulting in poor reliable target recognition. In future work the target recognition process must be improved by adding a decision procedure based on image features which are present in both synthetic and real ISAR images. Additionally, computational burden of other recognition methods based on the comparison between image features should also be studied in order to contrast results.

## Figures and Tables

**Table 1.** Target identification results

	***Synt.-B747***	***Synt.-Fokker 100***	***Synt.-B767***
***B-767 from actual data***	0.1927	0.2473	**0.3129**

**Table 2.** Operations needed to obtain an ISAR image.

**Stage**	**Substage**	**Number of Operations**	**% Time**
**HRRP Generation**	Windowing	N products of M complex samples	**3.24%**
	HRRP Formation	N IFFTs of M complex samples	**0.05%**
**Motion Compensation**	Range Bin Alignment	(N-1) circular correlation of M real samples	**86.81%**
	Scatterer Selection	N circular shifts	**0.39%**
	Phase Adjustment	N products of M complex samples	**0.10%**
**ISAR Formation**		M FFTs of N complex samples	**2.48%**

**Table 3.** Operations needed for range bin alignment.

**Real Products**	(N – 1)·M^2^
**Real Sums**	(N – 1)·M·(M – 1) + 5M·(N – 6)
**Shifts**	M·(N – 1) + 2(N – 1)
